# The effect of vaginal cylinder inhomogeneity on the HDR brachytherapy dose calculations using Monte Carlo simulations

**DOI:** 10.1002/acm2.14228

**Published:** 2023-12-03

**Authors:** Moeen Meftahi, William Y. Song

**Affiliations:** ^1^ Department of Radiation Oncology Virginia Commonwealth University Richmond Virginia USA; ^2^ Department of Radiation Oncology and Winship Cancer Institute Emory University Atlanta Georgia USA

**Keywords:** HDR brachytherapy dose calculations, Monte Carlo simulations, vaginal cylinder applicator heterogeneity

## Abstract

**Purpose:**

To analytically assess the heterogeneity effect of vaginal cylinders (VC) made of high‐density plastics on dose calculations, considering the prescription point (surface or 5 mm beyond the surface), and benchmark the accuracy of a commercial model‐based dose calculation (MBDC) algorithm using Monte Carlo (MC) simulations.

**Methods and materials:**

The GEANT4 MC code was used to simulate a commercial ^192^Ir HDR source and VC, with diameters ranging from 20 to 35 mm, inside a virtual water phantom. Standard plans were generated from a commercial treatment planning system [TPS—BrachyVision ACUROS (BV)] optimized for a treatment length of 5 cm through two dose calculation approaches: (1) assuming all the environment as water (i.e., D_w,w‐MC_ & D_w,w‐TG43_) and (2) accounting for the heterogeneity of VC applicators (i.e., D_w,w‐App‐MC_ & D_w,w‐App‐MBDC_). The compared isodose lines, and dose & energy difference maps were extracted for analysis. In addition, the dose difference on the peripheral surface, along the applicator and at middle of treatment length, as well as apical tip was evaluated.

**Results:**

The D_w,w‐App‐MC_ results indicated that the VC heterogeneity can cause a dose reduction of (up to) % 6.8 on average (for all sizes) on the peripheral surface, translating to 1 mm shrinkage of the isodose lines compared to D_w,w‐MC_. In addition, the results denoted that BV overestimates the dose on the peripheral surface and apical tip of about 3.7% and 17.9%, respectively, (i.e., D_w,w‐App‐MBDC_ vs D_w,w‐App‐MC_) when prescribing to the surface. However, the difference between the two were negligible at the prescription point when prescribing to 5 mm beyond the surface.

**Conclusion:**

The VCs’ heterogeneity could cause dose reduction when prescribing dose to the surface of the applicator, and hence increases the level of uncertainty. Thus, reviewing the TG43 results, in addition to ACUROS, becomes prudent, when evaluating the surface coverage at the apex.

## 1 INTRODUCTION

Vaginal cylinders (VC) are the most common applicator type used for high‐dose‐rate (HDR) brachytherapy (BT) in the patients suffering from endometrial cancer after surgery.^1^, ^2^, ^3^ Many of the commercial VCs are made of high‐density plastic materials, such as polyether ether ketone (PEEK) and polyphenylsulfone (PPSU) with a density of about 1.3 g/cc.^4^ Also, these applicators house at least one lumen inside for source positioning, which is filled with air. Therefore, these applicators create a heterogeneous environment around the HDR sources and could potentially influence the dose distributions, depending on the materials and their densities, the size, and the design of the VC applicator. In addition, the prescription point, either the surface or 5 mm beyond the surface of the VC, can magnify this effect.^5^ However, most of the currently available brachytherapy treatment planning systems (TPS) and clinical practice take advantage of the AAPM Task Group 43 (TG43) formalism to estimate dose inside the patients’ body using precalculated parameters obtained from single‐dose distributions in an infinite water medium. As a result, this method cannot consider the effect of shapes and materials other than water, such as VCs.^6^, ^7^, ^8^, ^9^, ^10^

To overcome these limitations, model‐based dose calculation (MBDC) algorithms have been developed and now are available in commercial TPSs. These algorithms rely on CT imaging of patients to account for scattering conditions different from that in the reference geometry for source dosimetric characterization, patient heterogeneities as opposed to a homogeneous water medium, and applicators.^11^ However, TG229 emphasizes on a need for reference dosimetry data obtained in liquid water phantoms to evaluate the uniform clinical implementation and robustness of these advanced dose calculation algorithms.^12^

The Monte Carlo (MC) simulations can be utilized for reference dosimetry and benchmarking the commercial MBDC algorithms, while accounting for the limitations related to the TG43 formalism for the absorbed dose calculations with a high accuracy.^10^, ^11^, ^13^, ^14^, ^15^, ^16^ In this study, we aim to assess the effect of VC heterogeneity on dose calculations and benchmark the accuracy of the commercial MBDC TPS (BrachyVision ACUROS (BV), Varian, Palo Alto, California, USA) against MC simulations. To this end, we performed a comprehensive study on a set of commercial universal VC applicators modeled in the BV TPS.

## 2 METHODS AND MATERIALS

The study comprises of two parts. The first part focuses on and explains the effect of heterogeneity only based on the MC simulations. The second part, in addition, benchmarks the accuracy of the BV TPS on dose calculations considering the effect of VC heterogeneity based on the two methods of prescription (normalization), namely, on the VC surface and 5 mm beyond the surface. For brevity, only the VC‐35 mm diameter was included in the second part of the study. Furthermore, the TG‐186 notations are used to distinguish between different methods of the dose calculations (i.e., MC vs. MBDC).^17^

### 2.1 Monte Carlo simulations

**FIGURE 1:**
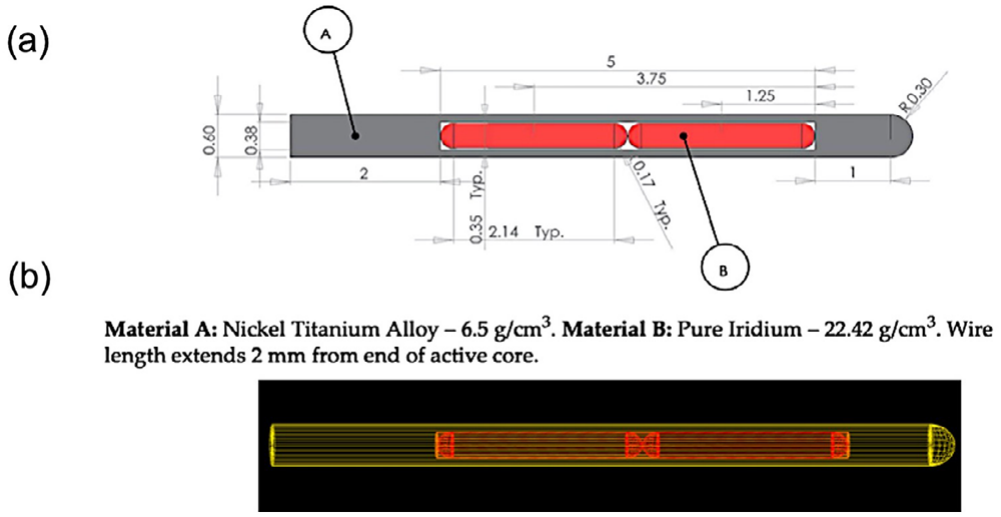
(a) 192Ir VS‐2000 source diagram from the BV algorithm reference guide 4. Unit is in [mm]. The white area inside the source is filled with air. (b) The simulated VS‐2000 (2012) source constructed using the GEANT4 MC code.

The GEANT4 MC package version 10.6 was utilized for all of the simulations. The Livermore physics with a cut value of 0.05 mm was picked for modeling the electromagnetic interactions. Detailed information about the GEANT4 MC package and its physics models can be found in the Conseil Européen pour la Recherche Nucléaire (CERN) documents and other literature.^18^, ^19^, ^20^, ^21^, ^22^ We simulated the VS‐2000 (Varian, Palo Alto, California, USA) ^192^Ir HDR brachytherapy source model as described in the BV algorithm reference guide.^4^ The G4UniounSolid was used to define the source geometry in detail. A diagram of the source structure and its simulation model are shown in Figure 1. For the TG43 parameters, including the 2D anisotropy factor, the radial dose function, and the dose rate constant, were obtained as described in the literature.^22^, ^23^, ^24^, ^25^

**FIGURE 2:**
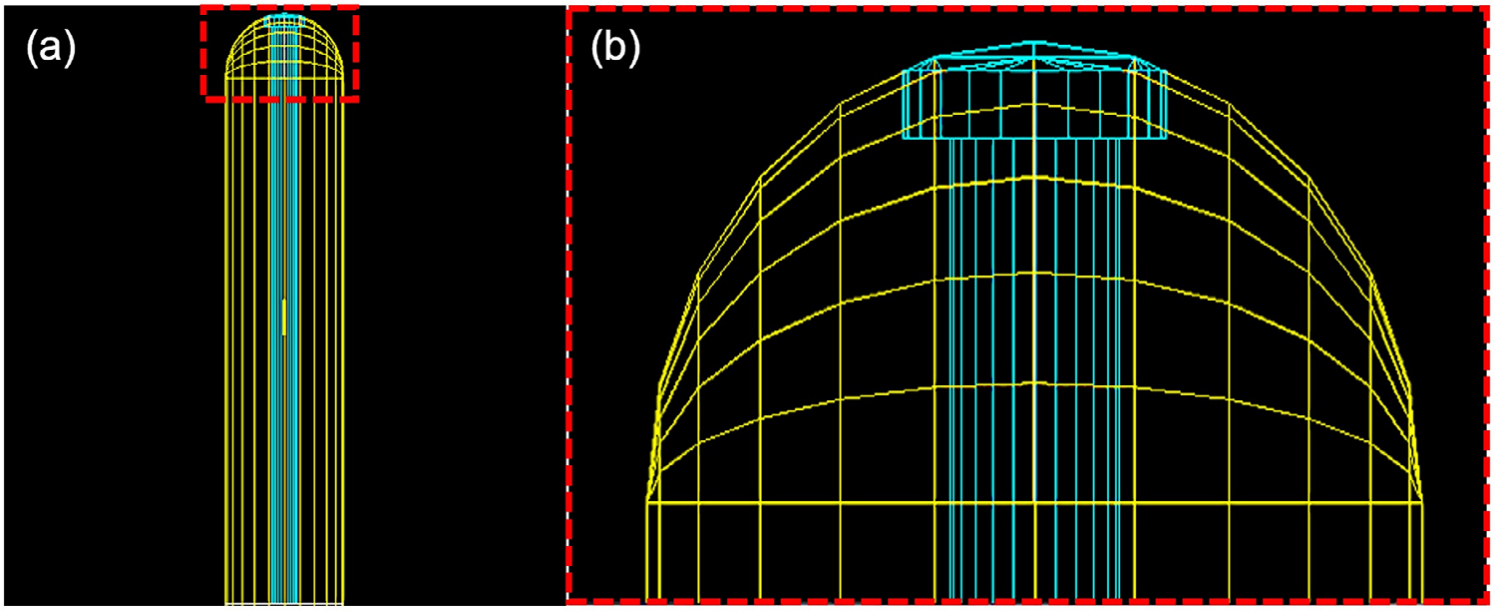
A simulated 30 mm VC applicator in GEANT4. (a) The wholistic view of the VC, including the PPSU outer cylinder shown in yellow, and the inner PEEK rod shown in blue. (b) A zoomed view of the apical part of the VC applicator shown in the red dash rectangle in (a).

For only‐MC heterogeneity study, we modeled the Varian's VC applicators, model 11011160, with diameters of 20, 25, and 30 mm. The applicators have two parts, including the outer tube made of PPSU plastic ([percentage weight: 95% H (4.0%), C (72.0%), O (16.0%), S (8%)] and a detachable universal inner tube made of PEEK plastic [percentage weight: 95% H (4.2%), C (79.1%), O (16.7%)] with densities of 1.30  and 1.31 g/cc, respectively.^4^ The inner part is 6 mm in diameter, has a dome shape piece at the top with a thickness of 3.13 mm, and includes a 1.5 mm diameter central lumen for an HDR source to travel through. The G4UnionSolid and G4Intersection were used to define the VCs’ geometry. A diagram of the simulated 30 mm diameter VC is shown in Figure 2.

We considered two different scenarios to assess the effect of heterogeneity on the dose calculations for a set of standard plans obtained from the BV TPS, prescribed to 5 mm beyond the VCs’ surface. The generation and optimization steps were as follows: 1) a treatment length of 5 cm was considered. 2) a prescription line displaced 5 mm from the surface of the applicator was drawn to cover the entire area of the interest (i.e., 5 cm treatment length). 3) dwell positions were incorporated in the lumen sequentially with a stepping length of 5 mm considering a 3 mm gap to the superior end of lumen for the first dwell position. 4) dwell times were inverse optimized using VEGO optimizer.

First, we performed simulations for when the VCs are inside a 30×30×30 cm^3^ virtual water phantom and accounted for the plastic materials and the single lumen (filled with air) heterogeneities (i.e., D_w,w‐App‐MC_). Second, we repeated the simulations with all the heterogeneities (i.e., the plastics and air in the lumen) as water to represent the TG43 conditions (i.e., D_w,w‐MC_). We ran the simulations for 4 × 10^9^ particle histories, and scored dose and energy deposited for each dwell position using a mesh with a voxel size of 0.5 mm. The statistical uncertainty for the GEANT4 data was less than 1%, on average, in the area of interest. Additionally, a separate grid set‐up was considered for the surface dose calculations to minimize statistical uncertainty. Due to symmetrical and cylindrical geometry, a cylindrical scoring was performed using co‐centric cylinders with an interval of 0.5 mm (i.e., the difference between the radius of each cylinder with the next one), starting from the central axis of VCs on an arbitrary plane crossing the applicators at Z = −3 cm. The height of each scoring volume was 0.5 mm, as well. None of the scoring volumes crossed the surface boundary of the applicators. The particle history for each dwell positions was also 3 × 10.^8^ Corresponding dose values at the surface and 5 mm beyond the surface were compared (i.e., GEANT4, D_w,w‐MC_ vs D_w,w‐App‐MC_).

### 2.2 BrachyVision ACUROS versus Monte Carlo simulations

In this study, the BV version 16.1 was used. The BV algorithm was developed to provide accurate and rapid dose calculations for HDR and pulsed‐dose rate (PDR) brachytherapy treatments.^4^ In the BV implementation, a Linear Boltzmann Transport Equation (LBTE) is deterministically solved using fine‐discretized grid of spatial, angular, and energy variables and then the average photon energy‐fluence distribution is obtained, which is then converted to a dose distribution.^26^ Further explanation about the BV algorithm can be found elsewhere.^4^, ^27^, ^28^, ^29^

For benchmarking of the BV, we used the VC with a diameter of 35 mm and provided the same set‐up condition (i.e., the same standard phantom, applicator set‐up, and grid size, voxel resolution, and set‐up) in both the GEANT4 MC simulations and the BV dose calculations. For BV, after creating a digital phantom with a slice thickness of 0.5 mm, the VC was inserted from the Solid Applicator library and placed along the Z‐axis, such that tip of the applicator coincided the origin. Further, we created two standard plans, as described above, for two normalization points: (1) the surface of the VC and (2) 5 mm beyond the surface of the applicator. A 3 mm gap for the first dwell position and a step length of 5 mm were also considered for each plan.

A grid size of 401×401×401 mm^3^ with a voxel size of (0.5 mm)^3^ was used for the dose calculations under two conditions: (1) considering all the heterogeneities (i.e., the plastics and air in the lumen) as water (TG43 conditions) (i.e., D_w,w‐TG43_ & D_w,w‐MC_), and (2) considering the effect of the VC heterogeneities (i.e., D_w,w‐App‐MBDC_ vs. D_w,w‐App‐MC_). We ran 4 × 10^9^ particle histories for MC simulations for each of the dwell positions inside the cylinder and scored the dose deposited. Additionally, dose on the peripheral surfaces along the length of the applicators (approximately) at the middle of treatment length were obtained by adapting the grid set‐up such that no voxels crossed the surface boundary. The average of the dose values over four voxels on a (an arbitrary) plane crossing the applicator at Z = −3 cm (i.e., X = 1.725 cm, X = −1.725 cm, Y = 1.725 cm, and Y = −1.725 cm) was considered as the peripheral surface dose. The dose at 5 mm beyond the surface was also determined similarly on the same plane. For MC simulations, in addition, the cylindrical scoring was performed as mentioned above. Corresponding dose values at the surface and 5 mm beyond the surface were compared for different approaches, including GEANT4 (D_w,w‐MC_ vs. D_w,w‐App‐MC_), BV (D_w,w‐TG43_ vs. D_w,w‐App‐MBDC_), and GEANT4 MC (D_w,w‐App‐MC_) vs. BV ACUROS (D_w,w‐App‐MC_).

### 2.3 Validation

In order to provide an independent approach to verify the heterogeneity effect on the surface dose, a point source estimation was performed. The source at each dwell positions were considered as a point source and the attenuation of ^192^Ir beam with an average energy 355 keV^12^ for given thicknesses of the water (TG43 conditions) and PEEK‐PPSU (plastic heterogeneity) was calculated at the point of interest on the surface at the plane Z = −3 cm. The cross‐sections were obtained from XCOM software.^30^ Further, the attenuation for each point source was weighted based on the corresponding dwell time. The weighted attenuations were further summed up to yield the total attenuation for each medium (i.e., water and plastic materials). Finally, the ratio of total attenuation in the plastic materials to water were considered as an estimation of dose difference in plastic materials versus TG43 (similar to D_w,w‐TG43_ vs. D_w,w‐App‐MBDC_, for example).

## 3 RESULTS

### 3.1 Monte Carlo simulations

A coronal view of the VS2000 (2012) ^192^Ir HDR source generated isodose lines and the 2D anisotropy function generated by the GEANT4 MC, compared to the consensus data from a previous study^12^, ^31^ are shown in Figure 1S (Supplement 1). We chose a 2% error bar on the GEANT4 MC data for the anisotropy function data points for display. As shown, there was an overall good agreement between the two sets of data.

Table 1S (Supplement 2) lists the numeric results between the GEANT4 MC and the consensus data^12^, ^32^ for the radial dose function, indicating a good overall agreement between the two. Furthemore, the dose rate constant obtained from the GEANT4 MC simulations, 1.109 ± 0.013 cGy/h/U, agreed well with the consensus data of 1.100 ± 0.006 cGy/h/U.^12^

**FIGURE 3:**
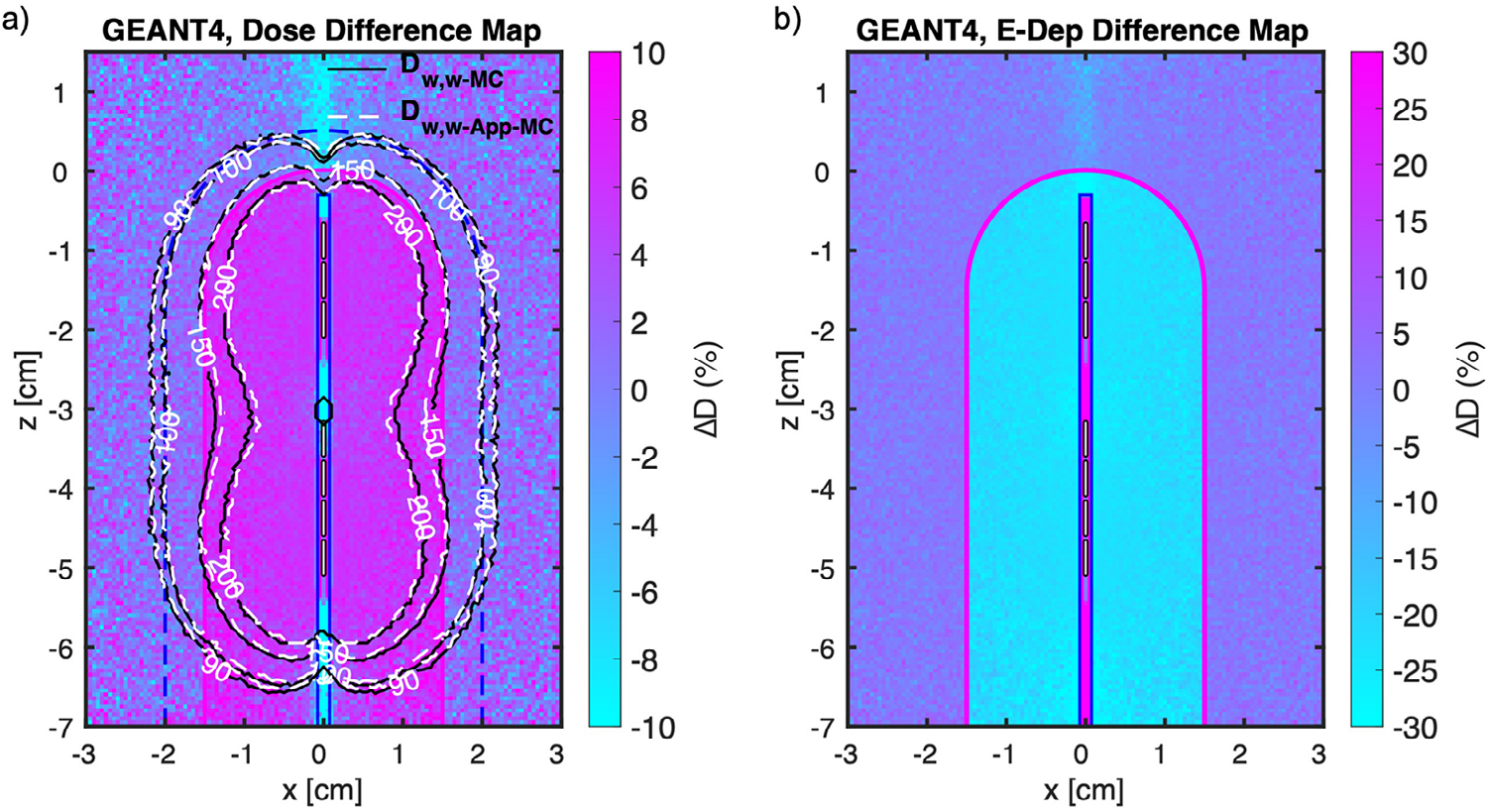
The outer contour of a 30 mm diameter VC applicator (solid magenta lines), along with the dwell positions (shown in white face) utilized to optimize the plan, is shown. (a) The compared isodose lines of D_w,w‐MC_ versus D_w,w‐App‐MC_ obtained from GEANT4 supported with dose difference map. The prescription line, which is 5 mm beyond the VC surface, is shown in dashed blue line. (b) The corresponding energy deposited difference map, as the difference in energy deposited for the D_w,w‐MC_ scenario compared to D_w,w‐App‐MC_ scenario. Note, the difference in energy deposited map (b) inside the high‐density plastics shown in cyan, indicating a notable increase in beam interactions inside the cylinder (up to 30% near the apical tip). However, the difference in energy deposited is negligible outside the cylinder in the water phantom.

The dose difference between (GEANT4) D_w,w‐MC_ versus D_w,w‐App‐MC_ models for different size of VCs based on the cylindrical scoring are given in Table 1. As presented, the dose values for D_w,w‐App‐MC_ models are imperceptibly (notably) smaller than the D_w,w‐MC_ models at the prescription point (the surface). The isodose lines and dose difference maps followed the same trend. Figure 3 visualizes the compared isodose lines supported with the dose and energy‐deposited difference maps for the two models for the 30 mm diameter VC applicator. The 100% isodose line indicates a dose of 2100 cGy (3 fxs × 700 cGy), prescribed to 5 mm beyond the surface. As shown, there is a shrinkage of the isodose lines by up to 1 mm for the D_w,w‐App‐MC_ results at the lateral periphery of the applicators, inside the VCs where the boundary of the water‐PPSU interface is located.

**TABLE 1:** The dose difference, D_w,w‐MC vs_ D_w,w‐App‐MC_, obtained from GEANT4, at the peripheral surface on a plane crossing the VC applicators at Z = −3 cm.

	∆D (%)	
	Surface	Surface + 5 mm
VC‐20 mm	5.9	1.2
VC‐25 mm	7.3	0.5
VC‐30 mm	7.4	0.1

The prescription point for this scenario is 5 mm beyond the surface. The differences are calculated at the surface and 5 mm beyond the surface of the VCs as given in the second and third columns.

As given in this example (i.e., Figure 3b), for all of the D_w,w‐App‐MC_ results, the energy deposition is significantly higher inside the VCs (up to 22% and 30% near the peripheral surfaces, along the length of the applicator excluding the curvature, and apical tip, respectively) than the corresponding D_w,w‐MC_ model. However, the opposite is true for the dose deposition, as shown in Figure 3a and presented in Table 1.

### 3.2 BrachyVision ACUROS versus Monte Carlo simulations

**FIGURE 4:**
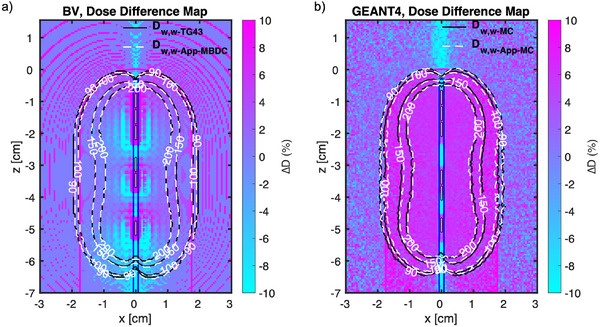
The outer contour of a 35 mm diameter VC applicator (solid magenta lines), along with the dwell positions (shown in white face) utilized to optimize the plan, is shown. The prescription line is the VC surface. (a) The compared isodose lines of D_w,w‐TG43_ and D_w,w‐App‐MBDC_ models obtained from BV supported with dose difference map. (b) The compared isodose lines of D_w,w‐MC_ and D_w,w‐App‐MC_ models obtained from GEANT4 supported with dose difference map. The dose difference level has been limited to [−10% +10%] to allow illustration of more details.

Figure 4 shows the compared isodose lines supported with dose difference map for the 35 mm diameter VC, comparing the BV algorithm and GEANT4 MC against TG43 conditions, respectively. The prescription point is at the surface of the applicator and the 100% isodose line indicates a dose of 2100 cGy (3 fxs × 700 cGy). The isodose lines from the TG43 protocol (D_w,w‐TG43_) and the ACUROS (D_w,w‐App‐MBDC_) extracted from BV algorithm highly coincide at the periphery (Figure 4a). However, compared to the D_w,w‐MC_ results the isodose lines from D_w,w‐App‐MC_ results show up to 1 mm of shrinkage starting just inside the applicator (Figure 4b). The maximum dose difference across the peripheral surface located between the fifth and the sixth dwell positions from the tip of the VC. Furthermore, both GEANT4 and BV results indicated a significant dose reduction (i.e., the blue streak) in TG43 models in the apical region along the lumen.

**FIGURE 5:**
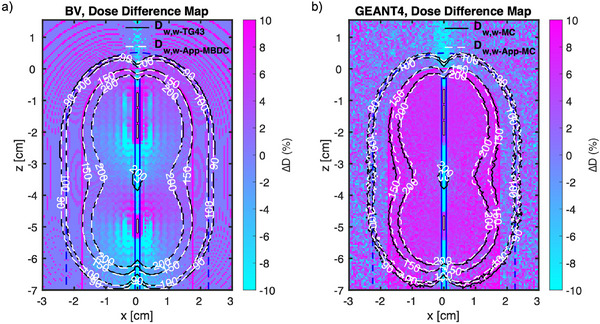
The outer contour of a 35 mm diameter VC applicator (solid magenta lines), along with the dwell positions (shown in white face) utilized to optimize the plan, is shown. The prescription line, which is 5 mm beyond the VC surface, is shown in dashed blue line. (a) The compared isodose lines of D_w,w‐TG43_ and D_w,w‐App‐MBDC_ models obtained from BV supported with dose difference map. (b) The compared isodose lines of D_w,w‐MC_ and D_w,w‐App‐MC_ models obtained from GEANT4 supported with dose difference map. The dose difference level has been limited to [−10% +10%] to allow illustration of more details.

Figures 5 illustrates the compared isodose lines supported with dose difference map for the 35 mm diameter VC, comparing the BV algorithm and GEANT4 MC against TG43 conditions, respectively. The prescription point is at 5 mm beyond the surface of the applicator and the 100% isodose line indicates a dose of 2100 cGy (3 fxs × 700 cGy). The isodose lines from the TG43 protocol (D_w,w‐TG43_) and the ACUROS (D_w,w‐TG43_) extracted from BV algorithm very much overlap at the periphery, overall (Figure 5a). However, compared to the D_w,w‐MC_ results the isodose lines from D_w,w‐App‐MC_ results show up to 1 mm of shrinkage starting just inside the applicator (Figure 5b). The maximum dose difference across the peripheral surface located between the third and the fourth dwell positions from the tip of the VC. Furthermore, both GEANT4 and BV results indicated a significant dose reduction (i.e., the blue streaks) in TG43 models in the apical region along the lumen.

Table 2 presents the dose difference comparison among different approaches, including BV (D_w,w‐TG43_ vs. D_w,w‐App‐MBDC,_ based on average results over four voxels), GEANT4 (D_w,w‐MC_ vs. D_w,w‐App‐MC,_ based on cylindrical scoring), and Point Source estimation (attenuation differences based on the XCOM cross‐sections). As given, there is a good agreement between GEANT4 and Point Source estimation. Also, there was some variation in D_w,w‐App‐MBDC_ versus D_w,w‐TG43_ in the results from BV approach, ranging from zero difference to up to ∼ 5%, as indicated by standard deviation (std).

**TABLE 2:** The dose difference comparison among different approaches on peripheral surface at the plane crossing the applicator length at z = −3 cm.

	BV		GEANT4		Point source estimation
	∆D (%)		∆D (%)		∆D (%)
Prescription point	Surface	Surface + 5 mm	Surface	Surface + 5 mm	Surface
Surface	2.3 ± 2.6	–	5.9 ± 0.0	–	5.9
Surface + 5 mm	2.7 ± 3.3	3.7 ± 2.2	6.2 ± 0.0	0.3 ± 0.0	6.1

The results for the BV and GEANT4 are given as (mean ± std) and (mean ± statistical uncertainty), respectively. The prescription point for this scenario includes both surface and 5 mm beyond the surface as indicated in the first column. The dose difference, ∆D (%), denotes D_w,w‐TG43_ versus D_w,w‐App‐MBDC_ for BV and D_w,w‐MC_ versus D_w,w‐App‐MC_ for GEANT4. In addition, ∆D (%) for the point source estimation approach implies the difference in the total attenuation of the beam in water versus high‐density plastics at the point of interest for the corresponding plans.

Table 3 provides direct comparison between BV (D_w,w‐App‐MBDC_, based on average results over four voxels) and GEANT4 (D_w,w‐App‐MC_, based on cylindrical scoring), as dose difference of ACUROS versus MC. As presented, the biggest difference between the two approaches would happen at the apical tip for when the prescription point would also be at the surface, where BV significantly overestimates the dose at the tip.

**TABLE 3:** The comparison between BV (D_w,w‐App‐MBDC_, based on average results over 4 voxels) and GEANT4 (D_w,w‐App‐MC_, based on cylindrical scoring) on peripheral surface, at the plane crossing the applicator length at z = −3 cm, and apical tip.

	∆D (%)			
	Peripheral surface		Apical tip	
Prescription point	Surface	Surface + 5 mm	Surface	Surface + 5 mm
Surface	3.5	–	17.9	–
Surface + 5 mm	1.0	0.0	10.7	‐1.6

The prescription point for this scenario includes both surface and 5 mm beyond the surface as indicated in the first column. The dose difference, ∆D (%) (D_w,w‐App‐MBDC_ vs. D_w,w‐App‐MC_), are given on the surface and 5 mm beyond the surface.

## 4 DISCUSSION

### 4.1 Monte Carlo simulations

All of the MC simulation results shown in this study (Table 1 and Figure 3) exhibited consistent dose reductions at the applicators’ surface (water‐PPSU boundary), compared to the TG43 protocol, with an average reduction of about 6.8% (Table 1). This can be better understood by examining the energy‐deposited and the dose difference plots as given in Figure 3a,b. We can see that the energy‐deposited is significantly higher inside the applicator (Figure 3b). This phenomenon arises from having more interactions of X‐ and gamma rays inside the applicators due to the higher density of the plastic materials (∼1.3 g/cm^3^) than that of water. Therefore, the bigger the applicator size, the higher the number of atoms and consequently the more interactions. This increases the energy deposition, of course, with up to 22% increase observed for the 30 mm diameter VC at the peripheral surfaces. However, this does not translate to increase in dose deposited inside the applicators because the increase in energy deposition is counteracted by about 30% higher mass of the plastics than water, where the dose is defined as energy deposited per unit mass [J/kg]. Therefore, as a result, we saw consistently less dose deposited, overall, inside the cylinders (Table 1). Since the x‐ray and gamma ray will interact and deposit their energies more in each layer of the plastic materials than water, the dose deposition will also occur at shorter distances from the source (dwell positions). Consequently, the shrinkage of the isodose lines will naturally occur (∼1 mm) and has been captured well by the MC simulations (Figure 3). The radiation fluence will also get slightly less intense after exiting the VCs, hence the dose values will decrease beyond the VCs’ surface (Table 1).

### 4.2 BrachyVision ACUROS versus Monte Carlo simulations

For the GEANT4 data (D_w,w‐MC_ vs. D_w,w‐App‐MC_), similar trends of dose deposition were observed in Figures 4 and 5 as compared to Figure 3. A major difference, however, is the additional variable of the prescription point's location (surface vs. 5 mm), which in turn affected the dwell positions’ locations as well, in order to generate optimal plans. In addition, the maximum difference occurred in the region where there were no dwell positions (e.g., between the fifth and sixth dwell position, Figure 4), due to the oblique filtration inside the applicator.

There were blue streaks in the apical region along the lumen applicators (Figures 4 and 5). This is because of the lumen, which is filled with air, and hence the X‐ and gamma interactions are much less compared with assuming all‐water by the TG43 protocol. Thus, the reduction of dose shown in the periphery of the VCs would be compensated for in the apex region, and hence the difference in dose is much smaller there. Also, it is important to note that the apex surface dose is about 35.3% and 32.2% less than the prescription dose, when prescribed to the surface and 5 mm, respectively. This is due to the filtration inside the iridium core and the capsule, leading to a well‐known anisotropic dip at the VCs’ tip (e.g., Figure 5). Since the central PEEK tube design is universal across all of the commercial VCs studied in this work, the results of the apical region should be similar for all VC sizes.

Based on the results presented, it is now apparent that the VCs’ heterogeneities can notably alter the dose distribution around the applicators, including the tip/apex and the lateral peripheral regions. This effect is more severe when prescribing to the applicators’ surface than at 5 mm distance (Table 1). The dosimetric disturbance effect, due to such high density plastics, also translates to a much higher dosimetric uncertainty level (up to 7.4% based on the MC results, Table 1) than the 1% set by the AAPM and GEC‐ESTRO guidelines.^33^ A good approach may be to reducing this uncertainty could be to use all of the available dwell positions and avoid large gaps between their positions when optimizing the plans.

The dose distributions generated by the BV algorithm (D_w,w‐TG43_ vs. D_w,w‐App‐MBDC_) showed minimal differences to TG43 in lateral periphery (Figures 4a and 5a) whether inside or outside of the VCs, within the area of clinical relevance (i.e., near the surface to 5 mm beyond the surface). The average difference in the region of interest was about 2.5 % (Table 2) regardless of the location of the prescription point. Such was not the case in apical direction (tip/apex), however, where large differences were observed in the water and PEEK regions. This, of course, is inconsistent with the GEANT4 comparison results (Figure 4a vs. Figure 4b), where in the water and PEEK regions, the two results are generally in agreement. The point source approach introduced in this study, in addition, verified the accuracy of the GEANT4 results. Thus, it may not be completely reliable to review the effects of heterogeneities with the BV algorithm alone, contrary to what others may claim^8^ and that MC simulations or other forms of reference dosimetry may be required, as emphasized by TG 229.^12^ To this end, Table 3 presented the direct comparison between the GEANT4‐ D_w,w‐App‐MC_ and the BV‐ D_w,w‐App‐MC_. The difference between the two results, inside the water, falls within 2% for both prescription points (surface vs. 5 mm) in the periphery. Although there is no clinical concern for the dose differences inside the applicators, the accurate estimation of the dose at the VCs’ surface would be concerning as it is a popular prescription/normalization point. Our analysis showed that the BV algorithm overestimates the dose up to 3.7% and 17.9% at the surface of the periphery and the apex regions, respectively, hence the potential for actual systematic under irradiation when prescribing to the surface exists (Table 3). In the case of the 5 mm prescription point, however, the differences between the two dose calculation techniques are near negligible (Table 3). In other words, the BV algorithm may not provide enough dosimetric accuracy at the boundaries of mediums. These overestimations could have also been introduced by the BV algorithm during the implementation of the discretization of the solution variables in space, angle, and energy.^4^ These sources could be the root causes of the discrepancies between the MC and BV algorithm results. Nonetheless, since the coverage of the apex is clinically concerning (site of the most recurrence), using the TG43 formalism (i.e., D_w,w‐TG43_ which underestimates the dose at the apex region) seems a prudent approach for the plan evaluation at this region when prescribing to the surface.

The study by Semeniuk et al.^34^ produced similar dose distributions and dose profiles for plastic materials as well as in water, using one dwell position inside a 36‐mm diameter VC. This study, however, did not expand to include cases with multiple dwell positions, which is more typical clinically. In addition, they scored the dose only outside the VC. Another study, by Petrokokkinos et al.,^28^ benchmarked the BV (version 8.8) against MC simulations for multiple dwell positions inside a 20 mm diameter VC, made of PMMA plastic (density = 1.19 g/cc), with a 180^o^ partial shielding. They studied the difference between the MC simulations and the TG43 protocol, as well. Although they showed some differences for MC versus TG43 as well as for MC versus BV using 2D colormaps, the scored volume for the dose calculations were still limited to the outside of the VC. Additionally, the presence of metal shielding on one side can affect the dose distribution in all scored volume due to a different scattering condition in the medium. Therefore, the differences reported cannot be attributed to the plastic heterogeneity alone. In contrast, the current study (1) provided a comprehensive dosimetry of the impact of the VC heterogeneity for ranging sizes of a commercial VC model, based on standard clinical plans (i.e., multiple dwell positions), (2) revealed how the prescription locations can contribute to the effect, (3) provided a detailed explanation of how VC heterogeneity could affect the dose calculations using dose‐ and energy‐ deposited maps, and (4) provided a site‐specific benchmark study (MBDCA vs. MC), which may further be available for correlation with known clinical outcomes.^35^

This study focused on one particular commercial universal VC design, from a single vendor (Varian, Palo Alto, California, USA), with a standard range of diameters (20‐35 mm), made from PEEK and PPSU plastic materials. Therefore, the results are limited to this commercial VC only, but perhaps translatable to other VCs using similar materials and designs. In addition, we only studied the dosimetric heterogeneity effect arising from the VCs. In the clinic, variables such as air bubbles, sutures, etc., could also be present in the treatment area.^36^, ^37^ Therefore, the impact of the heterogeneity effect on the target coverage could be more complicated, especially when prescribing to the surface. For the BV algorithm, careful evaluation in the apex/tip region would be prudent then as there are already multiple heterogeneities there (e.g., water, PEEK, air, etc.), along with frequent air bubbles and sutures (left in post hysterectomy) present within the treatment area. Further work to closely examine the effect of the combined heterogeneities of the VCs and air bubbles‐sutures could be valuable.

Finally, the type of the commercial VC design studied here (called the Stump VC) aims to provide maximum coverage at the vaginal apex region (the site of surgery). However, both the MC and BV (and even TG43) results showed large loss of coverage at the apex due to the anisotropy effect of the source design (∼30‐35% underdosage, e.g., see dosimetric dips in Figure 3). Such a loss of coverage is concerning but can be improved through a design modification via direction‐modulated brachytherapy (DMBT) concept approach, as recently published.^38^

## 5 CONCLUSION

We studied the effect of heterogeneities of high‐density plastic materials used for the fabrication of commercial VC applicators on dose calculations, using the GEANT4 MC and BV algorithm. The high‐density plastic material could cause a dose reduction on the peripheral surface of the cylinder and hence increase the level of uncertainty when prescribing the dose on the surface. However, this may not be crucial in the clinical setting, where positioning uncertainty, dose delivery, patient movement, etc., could pose a higher uncertainty.^33^ In addition, BV overestimate the dose at the boundary of the mediums, particularly at the apical tip. Therefore, utilizing the TG43 approach along with ACUROS would be wise, when evaluating the surface coverage at the apex using BV.

## AUTHOR CONTRIBUTIONS

Moeen Meftahi: Conceptualization, Methodology, Software, Validation, Formal analysis, Investigation, Writing—Original Draft, Visualization. William Y. Song: Conceptualization, Resources, Writing—Review and Editing.

## CONFLICT OF INTEREST STATEMENT

All other authors declare no conflicts of interest.

## Supporting information

Supporting Information

Supporting Information
