# Supplementary material for: The effect of vaginal cylinder inhomogeneity on the HDR brachytherapy dose calculations using Monte Carlo simulations
Source: J Appl Clin Med Phys. 2023 Dec 3;25(1):e14228. doi: 10.1002/acm2.14228 (PMC10795442; doi:10.1002/acm2.14228)
Supplement: Supplementary file 1 — Supporting Information [file ACM2-25-e14228-s002.docx]

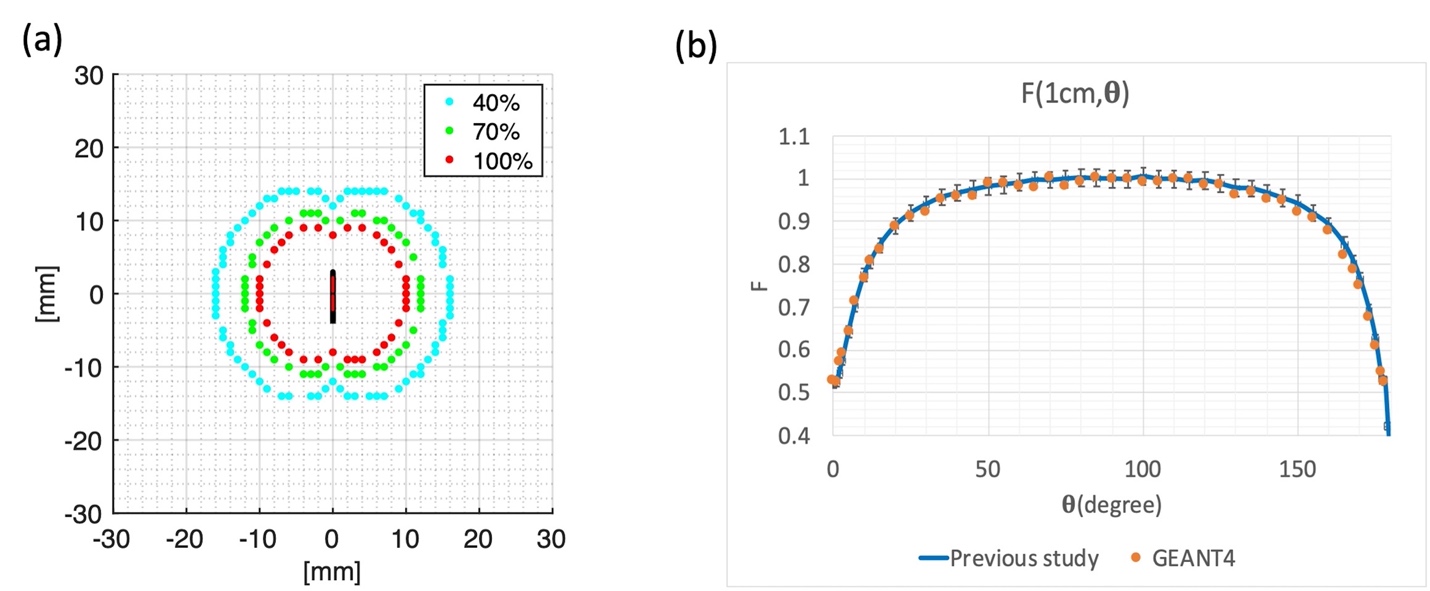


Figure 1S. (a) Isodose lines generated by the VS2000 (2012) source, in coronal view, derived from the GEANT4 MC results. Normalization point is 1 cm from the center of the Iridium core and perpendicular to the source axis. (b) The VS2000 (2012) 2D anisotropy function: comparison between a previous study versus GEANT4 MC results at r = 1 cm.
