# Supplementary material for: The effect of vaginal cylinder inhomogeneity on the HDR brachytherapy dose calculations using Monte Carlo simulations
Source: J Appl Clin Med Phys. 2023 Dec 3;25(1):e14228. doi: 10.1002/acm2.14228 (PMC10795442; doi:10.1002/acm2.14228)
Supplement: Supplementary file 2 — Supporting Information [file ACM2-25-e14228-s001.docx]

Table 1S. The VS2000 (2012) geometric function: comparison between the GEANT4 (G) and the previous study (PS) results.

| r(mm) | G | PS | G/PS |
| --- | --- | --- | --- |
| 2 | 0.99 | 0.98 | 1.01 |
| 5 | 0.99 | 0.99 | 1.00 |
| 7 | 0.99 | 0.99 | 1.00 |
| 10 | 1.00 | 1.00 | 1.00 |
| 15 | 0.99 | 1.00 | 0.99 |
| 20 | 1.00 | 1.01 | 0.98 |
| 30 | 1.00 | 1.01 | 0.99 |
| 40 | 1.01 | 1.01 | 1.00 |
| 50 | 0.99 | 1.01 | 0.98 |
| 60 | 0.98 | 1.00 | 0.98 |
| 80 | 0.97 | 0.98 | 0.99 |
| 100 | 0.95 | 0.95 | 1.00 |
